# In silico study of 14-3-3σ and Nipah virus W proteins interaction

**DOI:** 10.1038/s41598-026-57470-w

**Published:** 2026-06-11

**Authors:** Lia Griaznova, Vahram Arakelov, Grigor Arakelov, Karen Nazaryan

**Affiliations:** 1 Laboratory of Computational Modeling of Biological Processes, Institute of Molecular Biology of the National Academy of Sciences of the Republic of Armenia (NAS RA), 0014 Yerevan, Armenia; 2 Russian-Armenian University, 0051 Yerevan, Armenia

**Keywords:** 14-3-3 proteins, Nipah virus, Intrinsically disordered proteins, Protein–protein interactions, Molecular dynamics simulations, AlphaFold 3, Biochemistry, Biophysics, Computational biology and bioinformatics, Structural biology

## Abstract

Nipah virus (NiV) is a highly pathogenic zoonotic RNA virus that causes severe encephalitis, systemic vasculitis, and multiorgan damage in humans, with a case fatality rate of 40–75%. Among the viral factors implicated in NiV pathogenicity is the W protein, an intrinsically disordered product of the P gene. By binding to members of the 14-3-3 protein family, which regulate critical processes such as the cell cycle, apoptosis, and immune responses, W protein is thought to modulate host signaling pathways. Yet the molecular and structural basis of this interaction remains poorly understood. Here, we employed the AlphaFold 3 algorithm to predict 1000 models of the heterotetrameric complex formed by W protein dimers and the 14-3-3σ dimer. Candidate structures were filtered according to two structural criteria—potential disulfide bond formation between Cys421 residues of the W dimer, and preservation of interactions between phosphorylated Ser449 (Sep449) and residues Arg56, Arg129, and Tyr130 in the 14-3-3σ binding site. Nine models that satisfied these criteria were further refined and evaluated by molecular dynamics (MD) simulations in the OpenMM environment. Following 100-ns MD simulations, three models were selected for extended microsecond-scale simulations to assess their stability over extended timescales. The simulations indicated that all three complexes remained stable over the simulated timescales and displayed structural features consistent with interactions involving intrinsically disordered proteins. Among them, one model (Model 3) showed the most favorable combination of structural and energetic features. Our findings support the structural feasibility of the proposed interaction and demonstrate that the modeled heterotetrameric complex is capable of adopting stable conformations. These results provide a basis for further in silico studies and exploratory small-molecule screening aimed at modulating this protein–protein interaction in the context of viral host–pathogen interactions.

**Supplementary Information:**

The online version contains supplementary material available at 10.1038/s41598-026-57470-w.

## Introduction

The 14-3-3 protein family is a key regulator of diverse cellular processes, including cell cycle progression, apoptosis, and immune response, by recognizing phosphorylated motifs in target proteins^1^. Through their ability to engage in a wide range of protein–protein interactions, 14-3-3 proteins function as central hubs in cellular signaling networks^2^. Structurally, 14-3-3 proteins form dimers that create a conserved amphipathic groove, which serves as the primary binding site for phosphorylated partners^3,4^. In the 14-3-3σ isoform, this groove is lined with key residues such as Arg56, Arg129, and Tyr130, which stabilize phosphorylated motifs through electrostatic interactions and hydrogen bonding^5,6^. Depending on the sequence context, 14-3-3 proteins recognize their partners via several binding modes, including Mode III interactions involving C-terminal phosphosites, which are particularly relevant for viral proteins^5,7,8^.

Nipah virus (NiV), a highly pathogenic zoonotic paramyxovirus with case fatality rates reaching up to 75%, is classified as a biosafety level 4 agent and remains a significant global health concern^9,10^. Among its proteins, the W protein, produced via mRNA editing of the P gene, plays a key role in modulating host immune responses^11–14^. W is an intrinsically disordered protein (IDP) that contains a C-terminal phosphorylation site at Ser449, enabling interaction with host 14-3-3 proteins through a canonical recognition motif^15^. This interaction is believed to contribute to viral immune evasion by modulating host signaling pathways.

Although the ability of the W protein to bind 14-3-3 has been experimentally demonstrated, the structural organization of the full-length complex remains unresolved. This is largely due to the intrinsically disordered nature of W and the lack of experimental structural data for the complete assembly. To date, only a short W-derived peptide in complex with a single 14-3-3 binding groove has been structurally characterized^6^. In addition, previous studies suggest that the W protein may undergo dimerization via a disulfide bond involving Cys421 residues^16^, which could play a role in stabilizing the complex; however, the structural and functional implications of this process remain unclear.

In this study, we aimed to characterize the structural organization and interaction mechanism of the full-length W–14-3-3σ complex. To address this, we employed an integrative computational approach combining large-scale AlphaFold 3 modeling, structural filtering, and molecular dynamics simulations to generate and evaluate heterotetrameric assemblies formed by W and 14-3-3σ dimers. Our results support the structural feasibility of the proposed interaction and demonstrate that the complex can adopt stable conformations consistent with known binding features, highlighting key interactions associated with complex stabilization and providing a structural framework for future experimental validation.

**Fig. 1:**
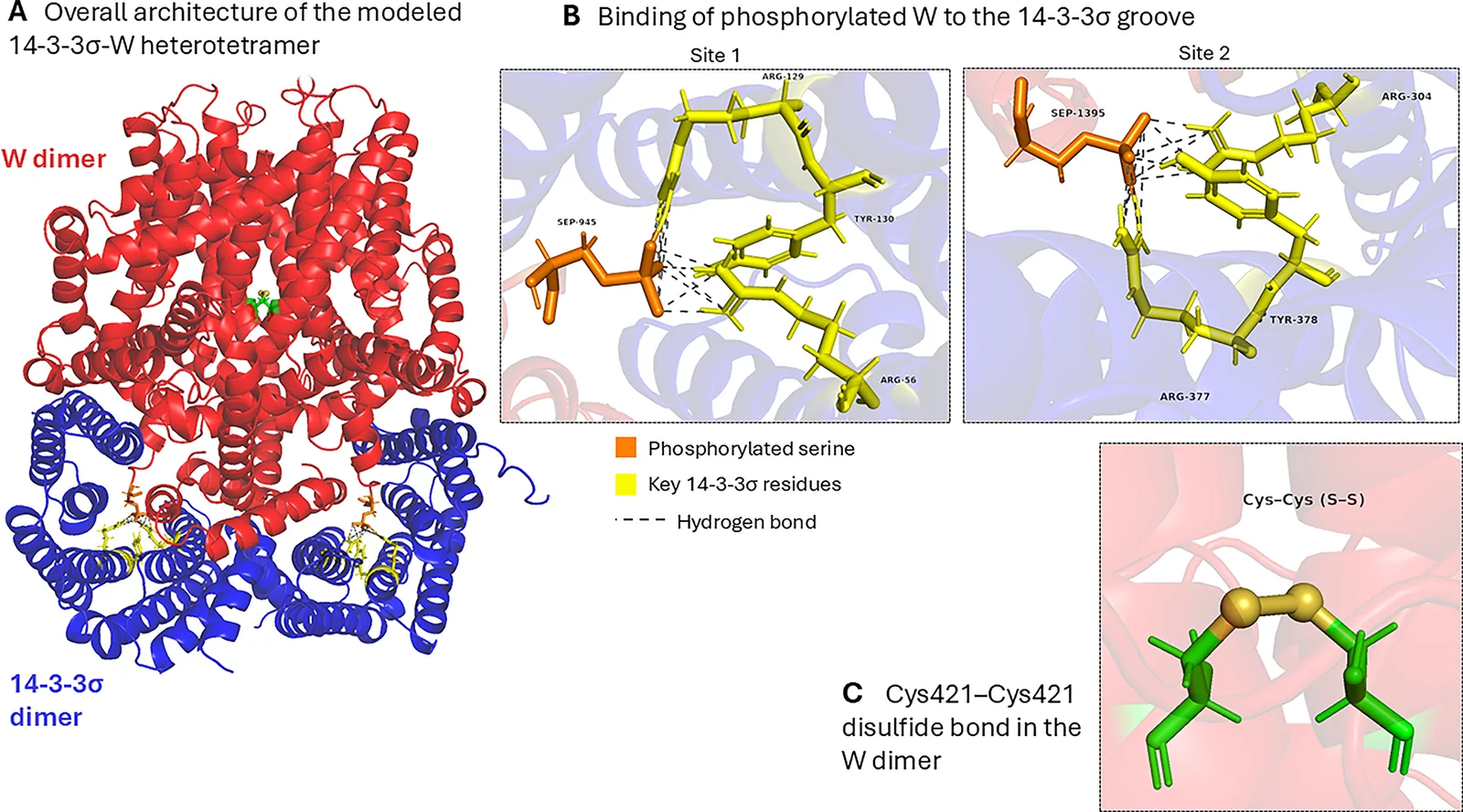
Structural organization of the 14-3-3σ–W complex. (**A**) Overall architecture of the modeled heterotetramer, showing the W protein dimer (red) and the 14-3-3σ dimer (blue). (**B**) Binding of phosphorylated Ser449 of the W protein to the 14-3-3σ binding groove at two interaction sites. Key residues (Arg56, Arg129, Tyr130) are shown in yellow, and hydrogen bonds are indicated by dashed lines. (**C**) Cys421–Cys421 disulfide bond stabilizing the W protein dimer.

**Fig. 2:**
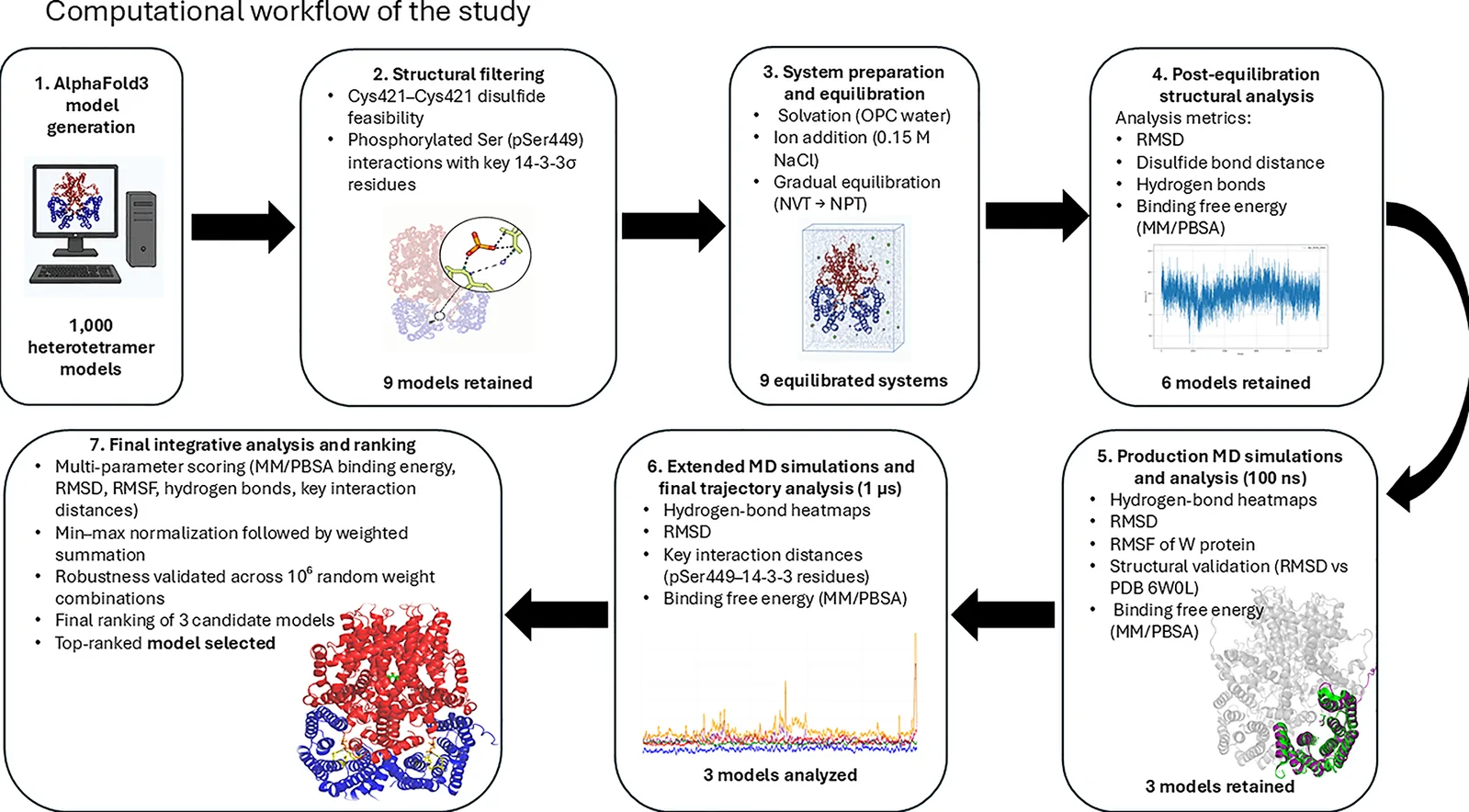
Computational workflow including AlphaFold 3 model generation, structural filtering, molecular dynamics simulations, and final model selection.

To facilitate structural interpretation of the modeled complexes, an overview of the heterotetrameric assembly is presented in Fig. 1, while a schematic representation of the computational workflow is shown in Fig. 2.

## Methods

Protein sequences of human 14-3-3σ (UniProt P31947) and Nipah virus W (UniProt P0C1C7) were used as input for AlphaFold 3 (web interface)^17^ to generate heterotetrameric assemblies consisting of a 14-3-3σ dimer and a W dimer. Post-translational modifications were enabled to account for phosphorylation of Ser449 in W. A total of 1000 independent runs with different random seeds were performed, and the resulting models were exported in mmCIF format for downstream analysis. A summary of the nine preselected models used for detailed analysis is provided in Supplementary Table S1, while the complete dataset of all 1000 generated models is available in the Supplementary Data.

Model selection was performed by calculating pairwise Euclidean distances between selected atoms according to two structural criteria: the S–S distance between Cys421 residues of the W monomers had to be ≤ 2.5 Å, indicating potential disulfide bond formation^18^, and the distance between Sep449 and the binding-site residues of 14-3-3σ (Arg56, Arg129, Tyr130) had to be ≤ 3.9 Å^19^. Donor–acceptor atom pairs for these interactions were defined based on the crystallographic 14-3-3σ-W peptide complex (PDB ID: 6W0L), where phosphate oxygens (O1P, O2P, O3P) of Sep449 interact with the guanidinium nitrogens (NH1, NH2) of Arg56 and Arg129 and the hydroxyl oxygen of Tyr130. Models meeting the disulfide criterion were prioritized even if Sep449 site distances slightly exceeded the threshold. As a result, nine models were retained for further MD simulations (Table S2). After equilibration, three models were excluded due to instability of the Cys421–Cys421 disulfide bond, and the remaining six were advanced to production simulations.

MmCIF files were converted to PDB format using VMD^20^, and system topologies were prepared with AmberTools (pdb4amber, tleap) using the ff19SB and phosaa19SB force fields^21^. The ff19SB force field was selected due to its improved backbone parameterization and more accurate description of secondary structure propensities compared to earlier Amber force fields. In combination with modern water models such as OPC, it provides a more balanced representation of protein–protein and protein–solvent interactions, which is important for modeling flexible and partially disordered systems^22^.

Systems were solvated in an octahedral OPC water box with a 10 Å buffer, neutralized, and adjusted to 150 mM NaCl^21^. MD simulations were performed in OpenMM^23^ with GPU acceleration (CUDA). After minimization and gradual equilibration, production simulations were run with a 4 fs time step, hydrogen-mass repartitioning (H = 1.5 amu), SHAKE constraints on bonds to hydrogen, PME electrostatics with a 1.0 nm cutoff, and a Langevin thermostat and barostat at 309.75 K and 1 atm^21–24^. Each of the six preselected models, representing independent starting conformations generated by separate AlphaFold 3 runs, was subjected to an independent MD simulation and simulated for 100 ns. Thus, comparative MD sampling was performed across multiple distinct starting conformations and equilibrated systems. The three most promising structures were subsequently extended to 1 μs.

During equilibration, the integration time step was 0.002 ps (2 fs), and coordinates were saved every 1000 steps, resulting in 5000 frames over 10 ns (0.002 ns per frame). In the subsequent production MD simulations, the integration time step was 0.004 ps (4 fs) with trajectory reporters triggered every 10,000 steps, producing 2500 frames over 100 ns and 25,000 frames over 1 μs (0.04 ns per frame). All trajectory plots are shown as a function of simulation time (ns).

Trajectory analysis was performed using cpptraj (AmberTools)^21^ and Python-based workflows. The analysis included measurement of the Cys421–Cys421 distance, per-frame estimates of binding free energies by MM/PBSA and MM/GBSA, hydrogen bond analysis for Sep449 with Arg56, Arg129, and Tyr130, distance measurements between Sep449 and the binding-site residues of 14-3-3σ, backbone RMSD of the complexes, and RMSF profiles for the W protein.

In addition, two independent 1 μs replicate simulations were performed for each of the three systems selected for extended simulations using different random initial velocities while maintaining identical simulation parameters. MM/PBSA binding free energy calculations were subsequently performed for all replicate trajectories to assess the reproducibility and energetic stability of the complexes.

Secondary-structure analysis was additionally performed using the DSSP algorithm implemented in cpptraj. The analysis was carried out for the W protein chains over the 100-ns MD trajectories, and the relative fractions of all major secondary-structure states (α-helix, 3–10/Pi helices, β-elements, turn/bend, and coil) were calculated for each frame. For comparative evaluation of the structural ordering across models, the time-dependent profiles of α-helical content were further analyzed using Python to obtain smoothed trajectories, while average secondary-structure distributions were calculated over the full trajectories.

Post-simulation models were also compared with the crystallographic 14-3-3σ-W peptide complex (PDB ID: 6W0L).

To rank the models, a composite scoring approach was implemented using a Python 3.10 workflow that integrated seven parameters: binding free energies (MM/PBSA), RMSD, RMSF, hydrogen-bond counts (in two chain pairs), and mean Sep449 binding-site distances (in two chain pairs). All parameters were min–max normalized, and the final score was calculated as a weighted sum. To assess the robustness of the ranking, additional tests were performed using 1000,000 random weighting schemes (drawn from a uniform distribution and normalized to sum to 1), ensuring that the selection of the best model was not dependent on a specific weighting scheme.

### Supplementary material

Supplementary material includes additional figures and tables describing model selection criteria, robustness analysis of the weighting scheme, and detailed molecular dynamics results (Figures S1-S3 and Tables S1–S7).

## Results

The models were classified into structured (< 0.3), intermediate (0.3–0.6), and disordered (> 0.6) groups according to previously reported criteria^25^, yielding distributions of 45.6%, 41.6%, and 12.8%, respectively (Table S1). The models selected for further analysis originated from different regions of this conformational space, indicating that the subsequent selection was not biased toward a single structural class. From this ensemble, nine models satisfying the predefined structural criteria were selected for detailed structural and energetic evaluation.

A comprehensive structural and energetic analysis was performed before MD simulations, resulting in the selection of Models 1–6 (from the preselected set of nine models) and the exclusion of Models 7–9. Comparison of disulfide bond RMSD, binding free energies (MM/GBSA and MM/PBSA), and atom counts after equilibration (Table S3) showed that Models 1–6 maintained stable Cys421–Cys421 disulfide linkages, with deviations not exceeding 0.05 Å from the reference length of ~ 2.05 Å. In contrast, Models 7–9 exhibited extended sulfur–sulfur distances, reaching up to 7.96 Å in Model 8, inconsistent with disulfide formation. Energetic calculations corroborated these observations: while most complexes displayed favorable negative binding free energies, Model 8 showed an abnormally high positive value, indicative of unstable binding. Heatmaps of hydrogen bonding (Figure S1) revealed that Sep449 formed the highest frequency of contacts with Arg56, Arg129, and Tyr130 in Models 3, 5, and 6. Global RMSD profiles (Figure S1) showed progressive stabilization of these models during equilibration. Models 7–9 were excluded from further consideration because they failed to maintain the critical disulfide bond, despite exhibiting other favorable parameters.

**Fig. 3:**
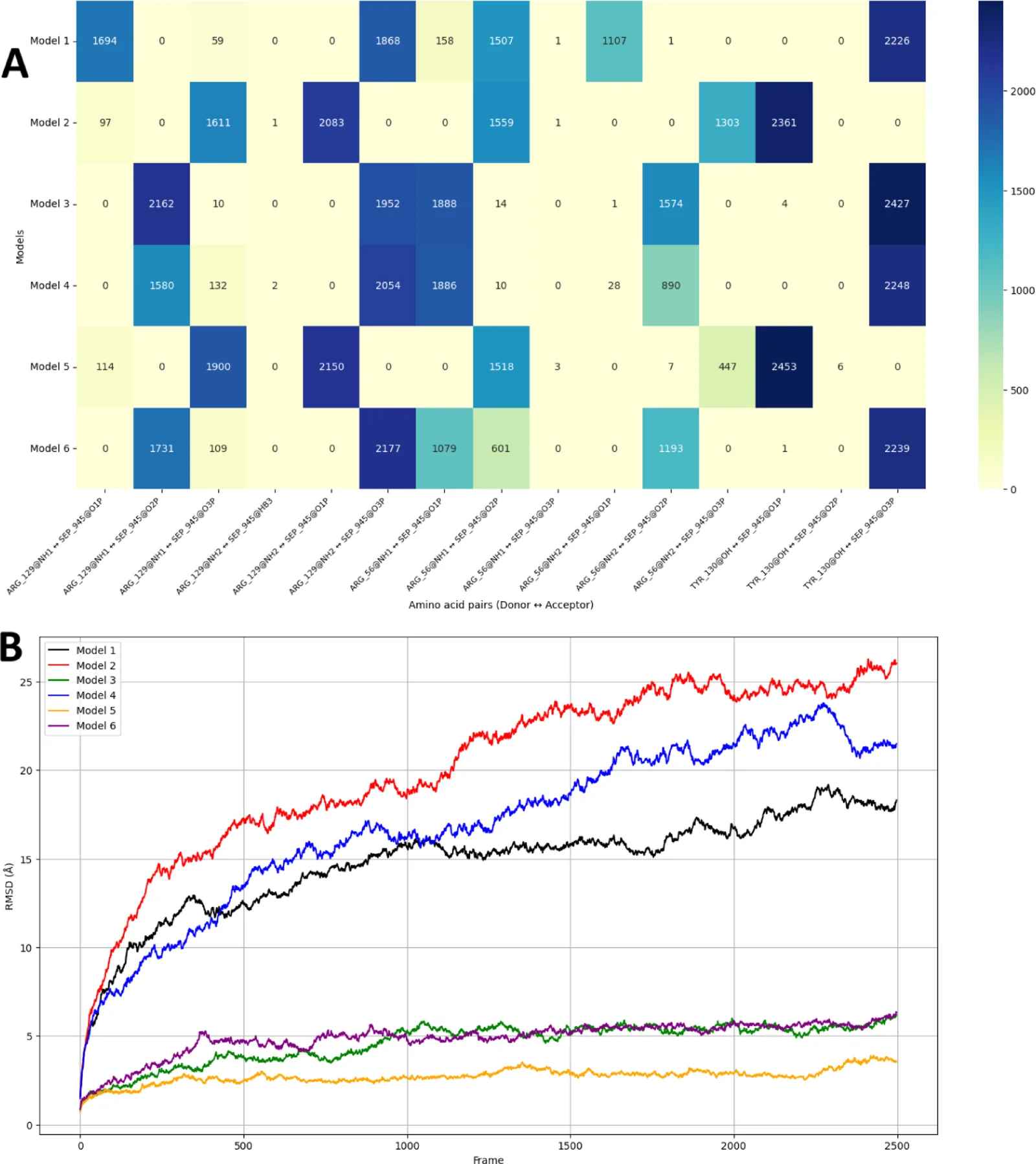
(**A**) Heatmap of atom-specific hydrogen bond frequencies between phosphate oxygen atoms of Sep449 and binding-site atoms of Arg56, Arg129, and Tyr130 in chains A–C for Models 1–6. The color scale represents the frequency of hydrogen bond formation, with darker shades indicating higher frequencies. (**B**) RMSD of 14-3-3–W complexes for different models during the 100-ns MD simulation.

All six preselected complexes (Models 1–6) were then subjected to 100-ns MD simulations. The disulfide bonds remained stable across all systems (Table S4). Hydrogen-bond analyses (Fig. 3) again highlighted Models 3, 5, and 6 as having more consistent and evenly distributed contacts between Sep449 and the 14-3-3σ binding site residues. Backbone RMSD traces (Fig. 3) further indicated that these three models tended to stabilize toward the end of the simulations, whereas Models 1, 2, and 4 continued to drift, suggesting less favorable conformational stabilization.

**Fig. 4:**
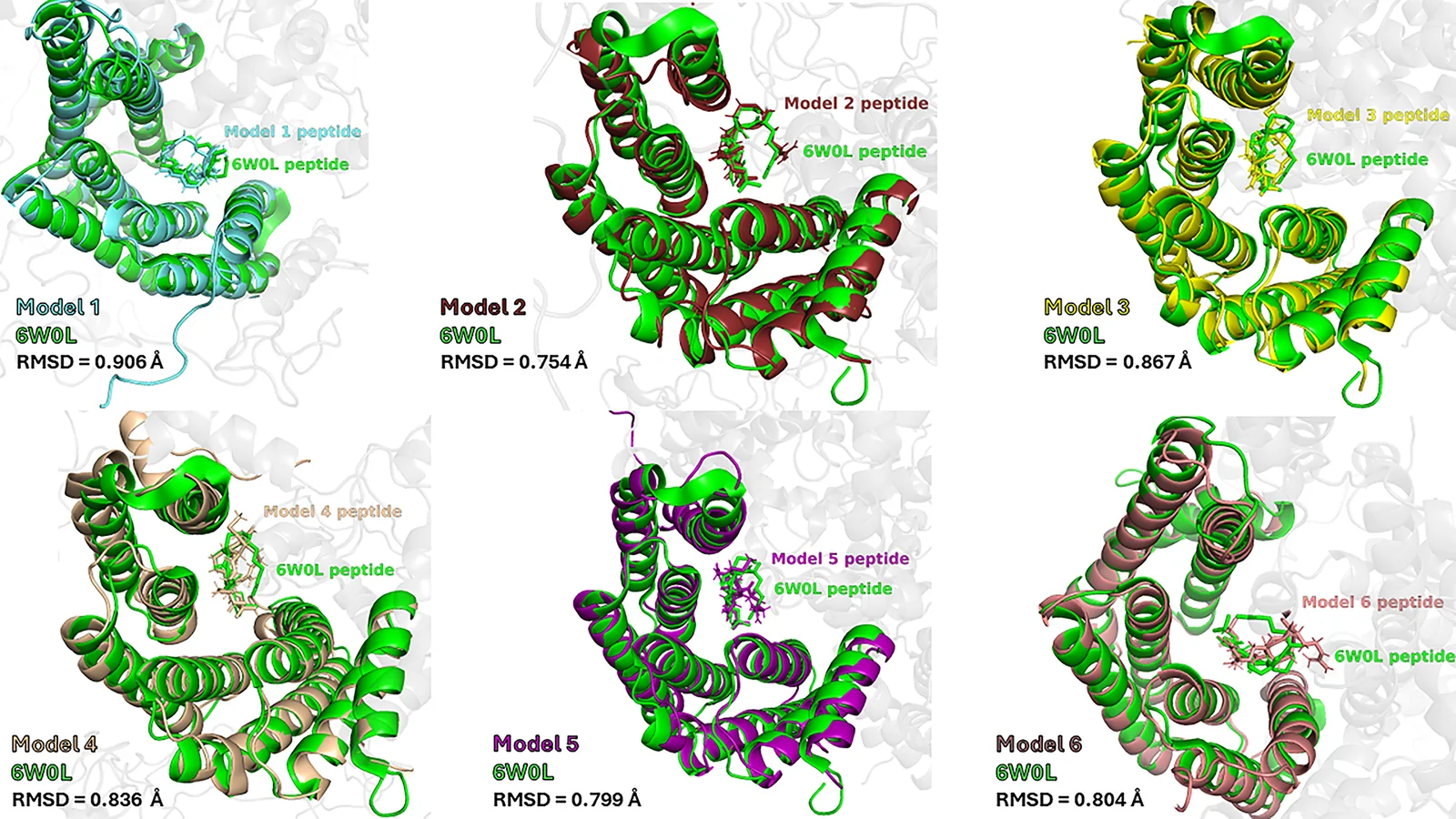
Structural alignment of six selected models of the 14-3-3σ–W protein complex with the reference crystallographic structure (PDB ID: 6W0L). The reference structure is shown in green, whereas the modeled complexes are depicted in distinct colors. Structural superposition was performed using chain A of 14-3-3σ together with the five-residue C-terminal fragment of the W protein corresponding to the experimentally resolved phosphopeptide motif. The obtained RMSD values (0.75–0.91 Å) indicate close correspondence of the modeled Sep449-containing interaction region with the crystallographic complex. Minor deviations reflect conformational variability in the positioning of the W peptide relative to the 14-3-3σ scaffold.

For additional structural validation, RMSD values were calculated by aligning the interacting region of each simulated complex with the crystallographic 14-3-3σ–W peptide complex (PDB ID: 6W0L). Specifically, the alignment was performed using chain A of the 14-3-3σ dimer together with the five-residue fragment of chain C of the W protein corresponding to the experimental binding motif. Superpositions were performed in PyMOL 3.1.6.1^26^ using the “align” command. The obtained RMSD values ranged from 0.75 to 0.91 Å (Fig. 4), indicating that the modeled Sep449-containing interaction region retained a close structural correspondence with the crystallographic reference complex.

**Fig. 5:**
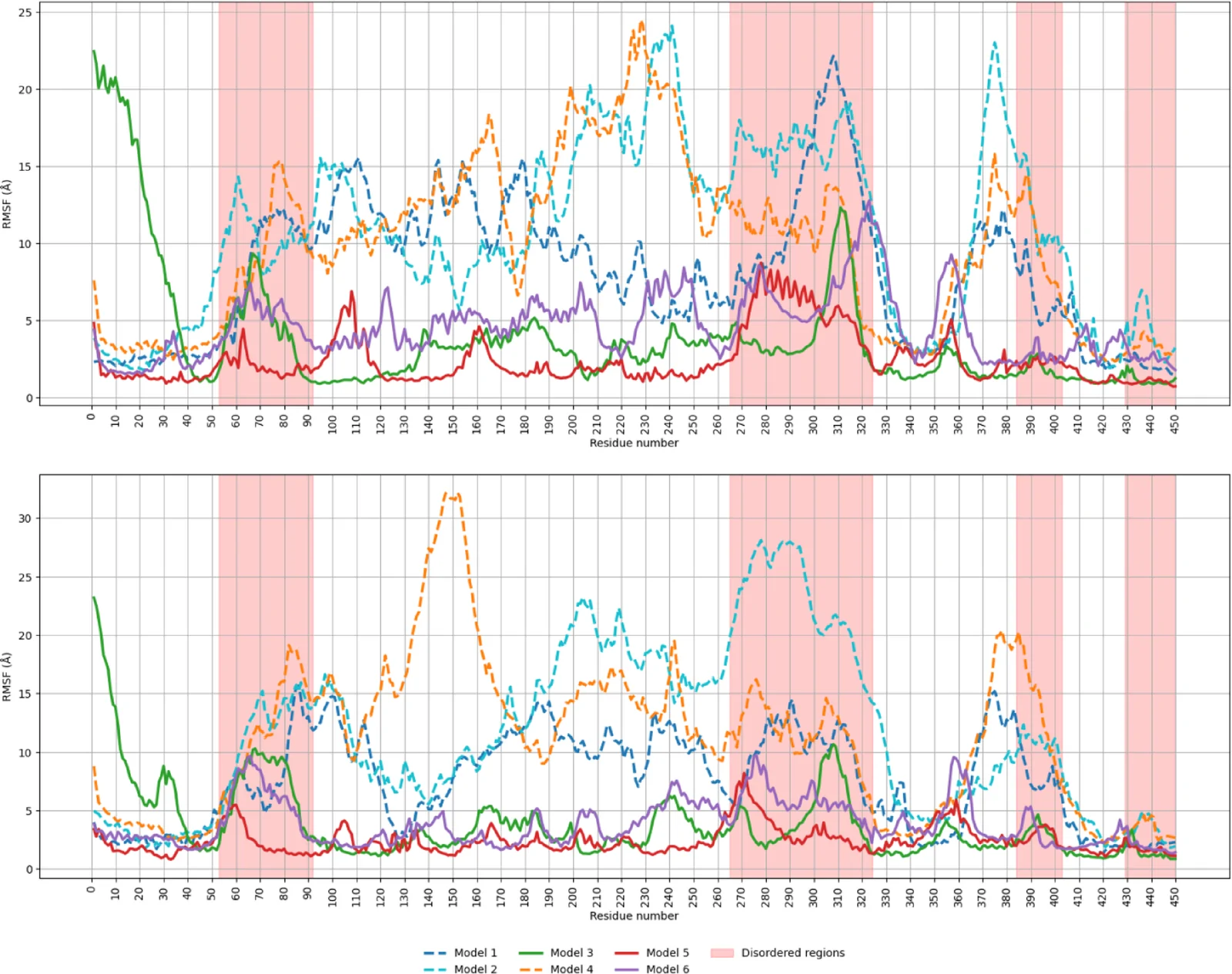
RMSF of W protein residues in chains C (top) and D (bottom) of the heterotetramer after 100-ns MD simulations. Disordered regions are highlighted in pink.

RMSF analysis of the W protein (Fig. 5) revealed that Models 3, 5, and 6 retained flexibility primarily within intrinsically disordered regions, whereas Models 1, 2, and 4 displayed more widespread fluctuations.

To further characterize the structural behavior of the W protein, secondary-structure analysis was performed using DSSP on the 100-ns MD trajectories (Figure S2). The analysis revealed a clear separation between two groups of models. Models 1, 2, and 4 were dominated by disordered coil/turn/bend states and exhibited consistently low α-helical content (~ 10–15%), indicating the persistence of highly flexible conformations throughout the simulations. In contrast, Models 3, 5, and 6 showed substantially higher α-helical content (~ 55–65%), which remained stable over time, while the remaining structural fraction was primarily represented by loop-like conformations. Notably, β-structural elements were negligible in all systems, indicating that the observed conformational differences are mainly associated with the degree of α-helical stabilization rather than alternative β-sheet formation.

Time-resolved analysis demonstrated that these differences persisted over the entire trajectory, suggesting the presence of distinct conformational states rather than transient fluctuations. Notably, Model 6 displayed a gradual decrease in α-helical content over time, indicating partial structural rearrangement during the simulation.

Taken together, these observations support the existence of a heterogeneous conformational ensemble of the W protein within the complex, where specific interaction modes promote partial structural stabilization. These observations provided additional structural support for advancing Models 3, 5, and 6 to extended simulations.

**Fig. 6:**
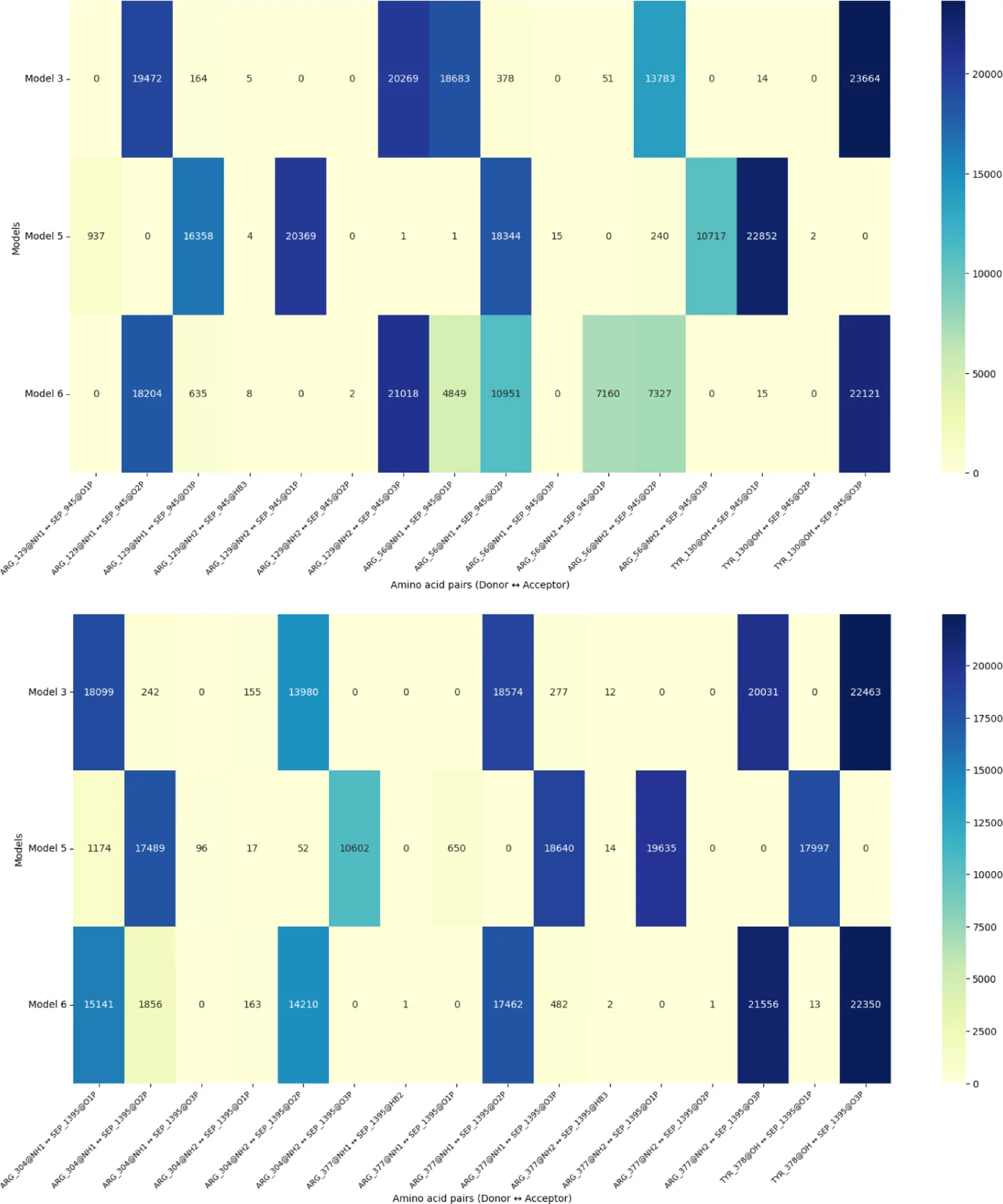
Heatmap of atom-specific hydrogen bond frequencies between phosphate oxygen atoms of Sep449 and binding-site atoms of Arg56, Arg129, and Tyr130 in chains A–C (upper panel) and B–D (lower panel) for Models 3, 5, and 6 after 1 µs MD simulation. The color scale represents the frequency of hydrogen bond formation, with darker shades indicating higher frequencies.

**Fig. 7:**
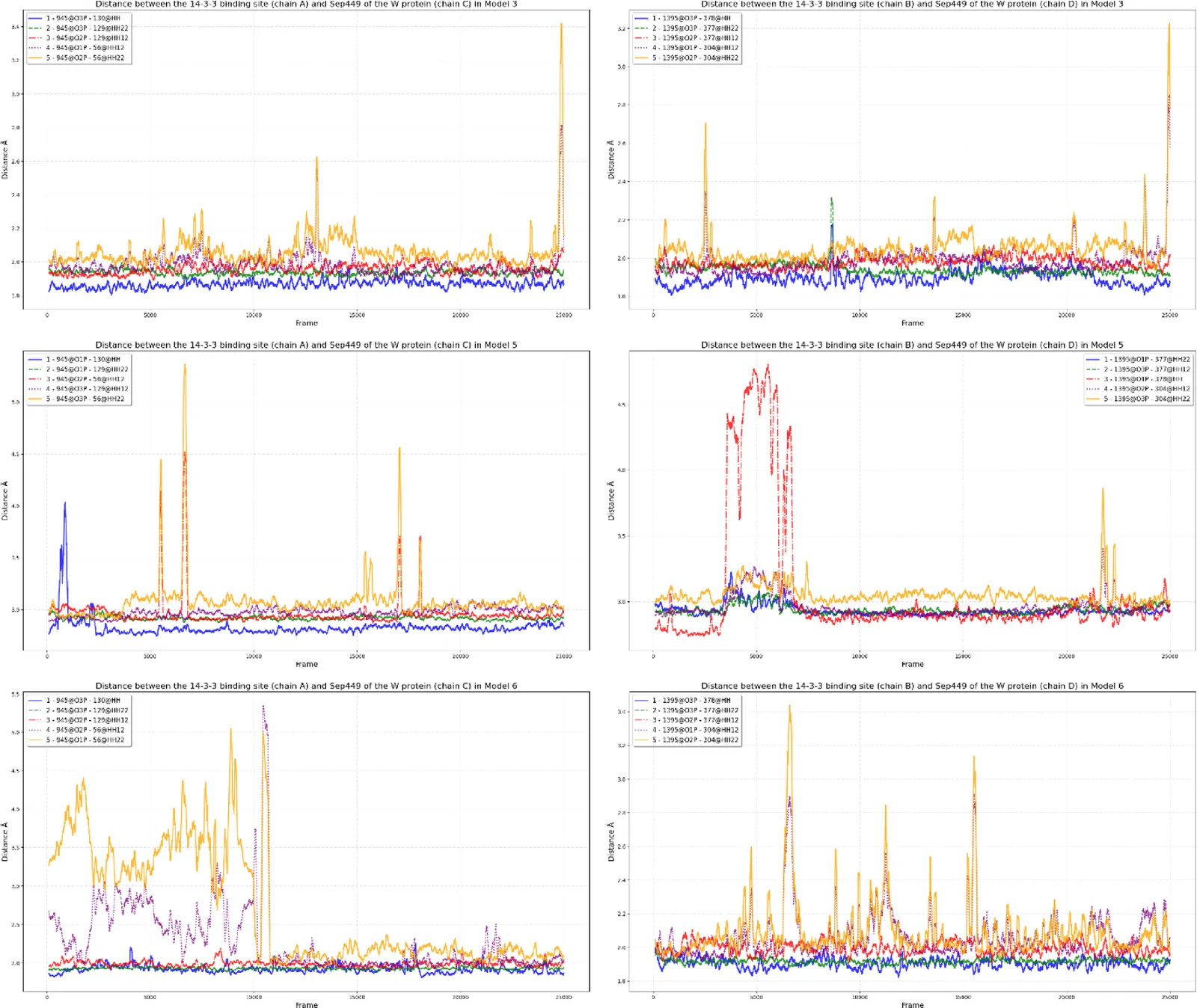
Distance between Sep449 of the W protein and the binding-site residues of 14-3-3σ in chains A–C (left panels) and B–D (right panels) for Models 3, 5, and 6.

**Fig. 8:**
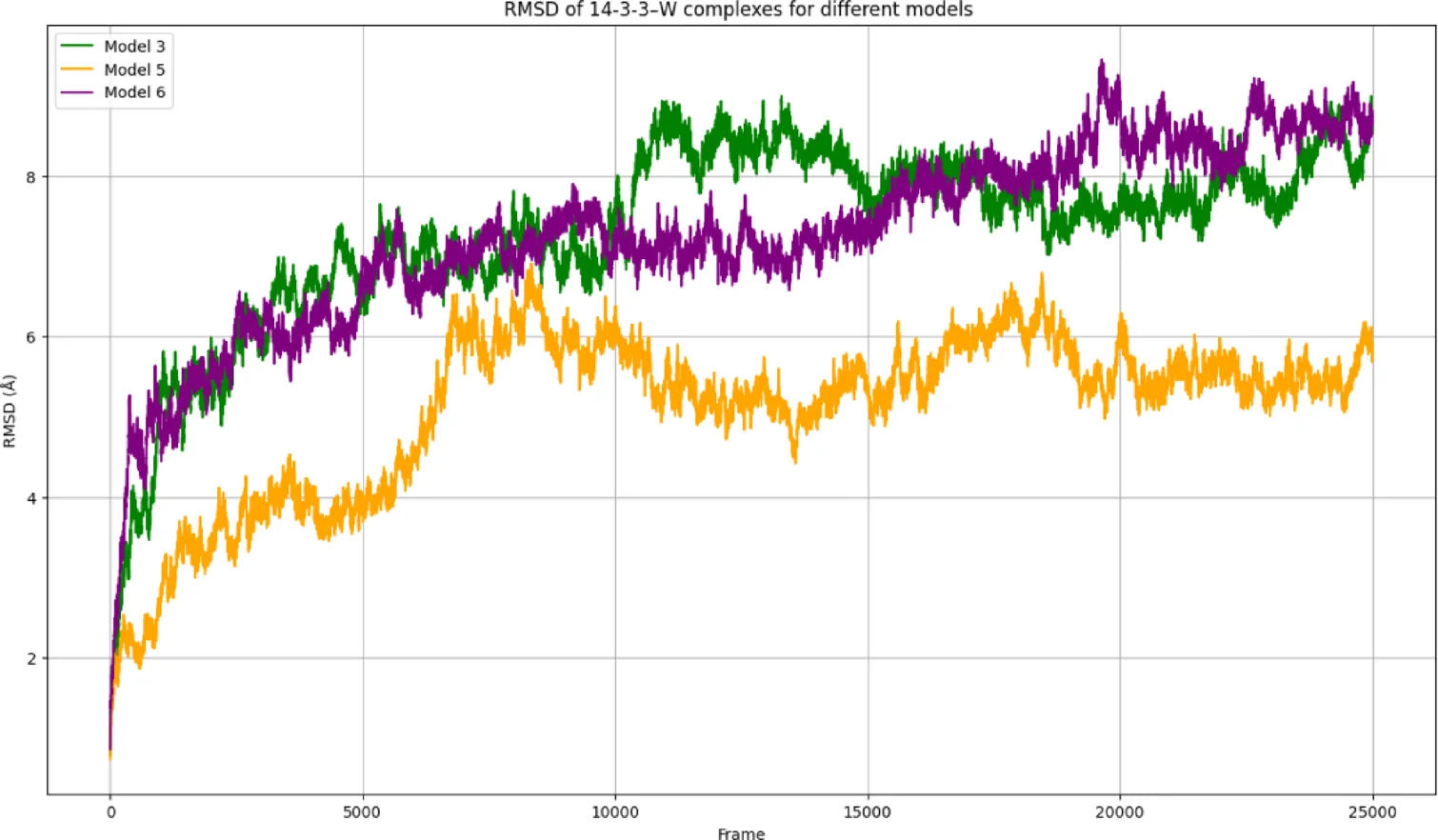
RMSD of 14-3-3–W complexes for different models after 1 µs MD simulation.

The three selected complexes were subsequently simulated for 1 µs to probe long-term stability, with the results summarized in Table S5–7 and Figs. 6–8. All models preserved stable disulfide bridges (RMSD ≤ 0.05 Å, Table S5). RMSF analysis of the binding site residues (Table S6) revealed restricted fluctuations (< 1.24 Å) in Models 3 and 5, while Model 6 exhibited greater flexibility, particularly at Arg129 (~ 2.05 Å). Heatmaps of hydrogen bonding (Fig. 6) confirmed persistent contacts between Sep449 and Arg56, Arg129, and Tyr130, with Tyr130 forming the highest number of stable bonds. Analysis of Sep449–site distances (Fig. 7) indicated that these interactions were most stable in Model 3, whereas Models 5 and 6 showed greater variability. Long-term RMSD profiles (Fig. 8) provide a general measure of global structural changes during the 1-µs simulations. Although RMSD is not an absolute criterion of stability, particularly for complexes containing intrinsically disordered regions such as the W protein, the observed values fall within the range typical of flexible protein systems. After ~ 300 ns, RMSD reached a plateau at 5–8 Å across all three models. Subsequent fluctuations remained moderate compared to the initial phase of the simulations, with Model 3 showing the smallest variation (~ 1.7 Å), followed by Models 5 (~ 2.0 Å) and 6 (~ 2.3 Å). Together with the persistent hydrogen-bond and distance patterns, these RMSD trends support the overall structural stability of the complexes under long-term simulation.

To further evaluate the reproducibility and energetic stability of the selected complexes, two additional independent 1 μs replicate simulations were performed for each model. MM/PBSA binding free energy calculations carried out for all replicate trajectories yielded highly consistent ΔG values across independent simulations (Figure S3). The average MM/PBSA energies and corresponding standard deviations confirmed the favorable energetic profiles of all three complexes. The observed variability of ΔG_total across independent trajectories remained limited, further supporting the reproducibility of the energetic behavior of the selected models. Together with the preservation of interprotein contacts, stable hydrogen-bond patterns, and limited variations in RMSD and RMSF, these results further support the long-term structural and energetic stability of the investigated complexes.

Finally, an integrative scoring approach was applied, combining binding free energies (MM/PBSA), RMSD, RMSF, hydrogen-bond counts, and key distance measures, using min–max normalization and weighted summation. To verify the robustness of this approach, 1000,000 random weighting schemes were tested, consistently identifying the same top-ranked model (> 99.9% of cases). For illustrative purposes, Table S7 presents results for seven arbitrarily chosen weighting schemes, demonstrating the stability of the ranking across different parameter combinations. Model 3 consistently achieved the highest composite scores, identifying it as the most stable and promising candidate for further study.

## Discussion and conclusions

In this study we performed the first comprehensive in silico investigation of the interaction between dimeric forms of the Nipah virus W protein and the human 14-3-3σ dimer. Using AlphaFold 3, we generated 1000 structural models of the heterotetrameric complex incorporating the phosphorylated residue Sep449. Nine models that met strict structural criteria—including the potential formation of a disulfide bond between Cys421 residues of the W dimer and stable contacts of Sep449 with Arg56, Arg129, and Tyr130 of 14-3-3σ—were selected for detailed analysis. Subsequent molecular dynamics simulations of 100 ns and 1 μs confirmed the persistence of the Cys421–Cys421 disulfide bridge and stable hydrogen bonds involving Sep449. Integrative evaluation across structural, energetic, and dynamic parameters consistently identified Model 3 as the most robust candidate, characterized by favorable binding energetics and conformational stabilization.

Our findings suggest that W protein dimerization, mediated by a disulfide bridge, may contribute significantly to the stabilization of its complex with 14-3-3σ. However, it should be noted that experimental evidence directly addressing the oligomeric state of the Nipah virus W protein remains limited. Therefore, the proposed disulfide-mediated dimerization should be considered a structural hypothesis derived from computational modeling rather than a confirmed biological mechanism. Moreover, the results indicate partial structural stabilization of regions of the intrinsically disordered W protein upon binding to 14-3-3σ. Rather than reflecting a strictly binding-induced folding process, this behavior is more consistent with the selection and stabilization of pre-existing conformations. This observation supports the hypothesis that conformational flexibility enables specific yet adaptable interactions with host proteins, allowing W to efficiently modulate cellular signaling processes.

To further interpret this behavior in the context of intrinsic disorder, according to UniProt annotation, approximately 31.6% of the W protein sequence is predicted to be intrinsically disordered, providing a baseline estimate of disorder based on sequence-derived features. However, AlphaFold3-derived models indicated variability in the predicted degree of disorder, as reflected by the fraction_disordered metric, suggesting that multiple conformational states may be accessible.

Importantly, the DSSP analysis supports the presence of heterogeneous secondary-structure behavior within the W protein across the analyzed complexes. While some conformations remained predominantly disordered, others exhibited partial α-helical stabilization, indicating that interaction with 14-3-3σ can promote different degrees of local structural ordering without inducing formation of stable β-structured domains.

Taken together, these observations suggest that the W protein is unlikely to adopt a single well-defined structural state, but rather exists as a heterogeneous conformational ensemble, where specific interaction modes with the 14-3-3 protein can promote varying degrees of structural ordering.

From a biological perspective, the modeled 14-3-3σ-W complex provides a plausible structural basis for experimental findings showing that W recruits 14-3-3σ to the nucleus and suppresses NF-κB-dependent transcription and interferon signaling^15^. The results suggest that W dimerization may enhance its affinity for 14-3-3σ, potentially stabilizing a multimeric assembly capable of sequestering the IκBα/p65 complex. Such interactions could underlie the mechanism by which Nipah virus suppresses proinflammatory gene expression and attenuates host immune responses.

The potential influence of the cellular environment, including factors such as ionic strength, macromolecular crowding, and subcellular compartmentalization, was not explicitly modeled in this study and represents an important direction for future investigation.

It should be noted that the present study is based entirely on in silico modeling and molecular dynamics simulations using modern force fields (ff19SB and phosaa19SB). Therefore, the results should be interpreted as computational predictions that outline possible molecular interaction mechanisms.

Beyond its biological implications, this study also highlights a methodological perspective: combining AlphaFold 3 and molecular dynamics can yield detailed structural hypotheses even for complexes containing flexible or partially disordered regions. The computational workflow—integrating model generation, structural filtering, long-timescale dynamics, and multi-parameter scoring—provides a reproducible framework for analyzing other protein–protein systems of biomedical relevance.

From an applied standpoint, the analysis focused on residues previously reported to be functionally important in the 14-3-3σ-W system. The residues Sep449, Arg56, Arg129, and Tyr130 are known to mediate phosphorylation-dependent interactions within the 14-3-3 binding pocket, whereas Cys421 has been implicated in W protein dimerization through potential disulfide bond formation. The present results support the structural stability of these interactions and emphasize their potential as reference points for the design of compounds capable of modulating or disrupting the 14-3-3σ-W interface. Such molecules could contribute to the development of antiviral strategies aimed at restoring normal 14-3-3 signaling and preventing NF-κB suppression.

In conclusion, the results of this study provide insights into the possible molecular basis of 14-3-3σ–mediated regulation during Nipah virus infection. The findings suggest that dimerization and phosphorylation may jointly contribute to the stabilization of the 14-3-3σ-W complex and influence its dynamic behavior. The reproducibility of the observed structural and energetic trends was further supported by independent replicate simulations and MM/PBSA calculations, which yielded consistent results across all trajectories of the selected models. These additional analyses increase confidence in the robustness of the proposed interaction models and the conclusions derived from the molecular dynamics simulations. Overall, this work provides a computational framework that could support future structural and biochemical investigations aimed at exploring these interactions and assessing their potential therapeutic relevance.

## Supplementary Information

Supplementary Information 1.

Supplementary Information 2.

Supplementary Information 3.

Supplementary Information 4.

Supplementary Information 5.

## Data Availability

All data generated or analyzed during this study are included in this published article and its Supplementary Information files. Structural models generated in this study, including the final selected model, are available from the corresponding author upon reasonable request.
