# Supplementary material for: In silico study of 14-3-3σ and Nipah virus W proteins interaction
Source: Sci Rep. 2026 Jun 11;16:26550. doi: 10.1038/s41598-026-57470-w (PMC13503744; doi:10.1038/s41598-026-57470-w)
Supplement: Supplementary file 1 [file 41598_2026_57470_MOESM4_ESM.docx]

**Supplementary Material**

**Figure S1.** (A) Heatmap of atom-specific hydrogen bond frequencies between phosphate oxygen atoms of Sep449 and binding-site atoms of Arg56, Arg129, and Tyr130 in chains A–C across the preselected models after equilibration. The color scale represents the frequency of hydrogen bond formation, with darker shades indicating higher frequencies. (B) RMSD of 14-3-3–W complexes for different models after equilibration.

**Figure S2.** (A) Time evolution of α-helical content of the W protein across six models during 100-ns MD simulations. Smoothed trajectories (rolling average) are shown. Models 3, 5, and 6 exhibit consistently higher α-helical content compared to Models 1, 2, and 4. (B) Average distribution of major secondary-structure states in the W protein over the 100-ns trajectories, including α-helices, coil/other, turn/bend, 3-10/Pi helices, and β-elements.

**Figure S3.** Reproducibility of MM/PBSA binding free energy calculations across independent 1-μs molecular dynamics simulations of the selected complexes. Panels A–C show the distributions of total binding free energy (ΔG_total) values obtained for three independent trajectories of Models 3, 5, and 6, respectively. For each replicate, the mean value, standard deviation (SD), and standard error of the mean (SEM) are indicated. Panel D compares the average ΔG_total values for all models, calculated across the three independent simulations, with error bars representing standard deviations. The results demonstrate good agreement between replicate simulations and confirm the reproducibility of the energetic stability trends observed for the selected complexes.

| **Seed** | **Model_ID** | **Ranking_score** | **Fraction_disordered** | **Category** | **Disulfide_7132-10609** | **435-7360** | **436-7362** | **1022-7362** | **1023-7361** | **1035-7361** | **Model in article** |
| --- | --- | --- | --- | --- | --- | --- | --- | --- | --- | --- | --- |
| 909291325 | 4 | 0,540 | 0,050 | Structured | 2,463 | 4,114 | 3,785 | 4,642 | 2,734 | 2,912 | 8 |
| 182536966 | 4 | 0,580 | 0,060 | Structured | 2,200 | 3,925 | 3,158 | 2,391 | 5,024 | 4,218 | 5 |
| 983746281 | 4 | 0,570 | 0,060 | Structured | 2,101 | 2,732 | 4,505 | 3,386 | 3,822 | 4,529 | 3 |
| 450 | 3 | 0,600 | 0,080 | Structured | 2,202 | 2,550 | 4,407 | 3,460 | 3,703 | 4,633 | 6 |
| 860158376 | 3 | 0,800 | 0,540 | Intermediate | 2,012 | 3,675 | 4,561 | 3,530 | 5,237 | 4,036 | 1 |
| 1887236744 | 0 | 0,820 | 0,580 | Intermediate | 2,153 | 2,847 | 4,428 | 3,452 | 3,831 | 4,643 | 4 |
| 1417186589 | 1 | 0,820 | 0,580 | Intermediate | 2,486 | 3,905 | 3,602 | 4,911 | 3,985 | 4,852 | 9 |
| 144377815 | 0 | 0,850 | 0,620 | Disordered | 2,059 | 3,969 | 2,981 | 2,801 | 5,249 | 4,110 | 2 |
| 1961175291 | 1 | 0,850 | 0,650 | Disordered | 2,304 | 3,814 | 4,521 | 3,431 | 5,120 | 3,870 | 7 |

**Table S1.** Summary of structural characteristics of the nine preselected AlphaFold3 models of the 14-3-3σ–W protein complex. The full set of 1,000 generated models was used to assess structural diversity, and the complete dataset is provided in the Supplementary Data.

Each model is identified by its original AlphaFold3 seed and model index. For clarity, the models are numbered from 1 to 9 in the main text (see column “Model in article”). For each structure, the ranking score, fraction of disordered residues, and structural classification are provided. Distances corresponding to the Cys421–Cys421 disulfide bond (7132–10609) and key interactions between Sep449 and residues Arg56 (435–7360, 436–7362), Arg129 (1022–7362, 1023–7361), and Tyr130 (1035–7361) of 14-3-3σ are reported in Å.

Across the complete set of 1,000 generated models, the distribution of structural categories was as follows: Structured (<0.3): 458 models (45.6%); Intermediate (0.3–0.6): 418 models (41.6%); Disordered (>0.6): 129 models (12.8%).

| Model | C421 & C421 (W protein disulfide bonds)  Distance (Å) | R56 (14-3-3) - Sep449 (W)  Distance (Å) | | R129 (14-3-3) - Sep449 (W) Distance (Å) | | Y130 (14-3-3) - Sep449 (W) Distance (Å) |
| --- | --- | --- | --- | --- | --- | --- |
|  |  | NH1–O1P | NH2–O3P | NH1–O3P | NH1–O2P |  |
| Model 1 | 2.017 | 3.68 | 4.56 | 3.53 | 5.24 | 4.04 |
| Model 2 | 2.06 | 3.97 | 2.98 | 2.8 | 5.25 | 4.11 |
| Model 3 | 2.1 | 2.73 | 4.51 | 3.39 | 3.82 | 4.53 |
| Model 4 | 2.15 | 2.85 | 4.43 | 3.45 | 3.83 | 4.64 |
| Model 5 | 2.2 | 3.92 | 3.16 | 2.39 | 5.02 | 4.22 |
| Model 6 | 2.2 | 2.55 | 4.41 | 3.46 | 3.7 | 4.63 |
| Model 7 | 2.3 | 3.81 | 4.52 | 3.43 | 5.12 | 3.87 |
| Model 8 | 2.46 | 4.11 | 3.79 | 4.64 | 2.73 | 2.91 |
| Model 9 | 2.49 | 3.9 | 3.6 | 4.91 | 3.98 | 4.85 |

**Table S2.** Structural parameters of the selected models: C421–C421 disulfide bond distances and hydrogen-bond distances between Sep449 (W) and Arg56/Arg129/Tyr130 (14-3-3σ), measured using donor–acceptor atom pairs derived from the crystal structure (PDB 6W0L).

| Model | RMSD disulfide bonds | Binding Free Energy of the Complex | | Total atom count |
| --- | --- | --- | --- | --- |
|  |  | GB | PB |  |
| Model 1 | 0.05 | -232.11 | -306.51 | 504 808 |
| Model 2 | 0.05 | -145.09 | -310.17 | 620 890 |
| Model 3 | 0.04 | -439.69 | -305.80 | 91 106 |
| Model 4 | 0.04 | -264.50 | -318.29 | 503 732 |
| Model 5 | 0.05 | -261.96 | -292.24 | 146 876 |
| Model 6 | 0.05 | -239.91 | -315.77 | 111 172 |
| Model 7 | 2.75 | -245.45 | -305.39 | 415 914 |
| Model 8 | 7.96 | 12416.50 | 13459.73 | 90 462 |
| Model 9 | 3.46 | -219.21 | -262.21 | 469 274 |

**Table S3.** Comparison of structural and energetic parameters for nine AlphaFold 3-generated models after equilibration. Parameters include RMSD of the Cys421–Cys421 disulfide bond (Å), binding free energy of the complex calculated by MM/GBSA (GB) and MM/PBSA (PB) methods (kcal/mol), and total atom count in the system.

| Model | RMSD disulfide bonds | Binding Free Energy of the Complex | | Total atom count |
| --- | --- | --- | --- | --- |
|  |  | GB | PB |  |
| Model 1 | 0.05 | -235.2856 | -260.1758 | 504 808 |
| Model 2 | 0.05 | -260.0540 | -339.7707 | 620 890 |
| Model 3 | 0.04 | -358.2724 | -337.9479 | 91 106 |
| Model 4 | 0.04 | -275.7104 | -321.1254 | 503 732 |
| Model 5 | 0.05 | -188.5644 | -219.7187 | 146 876 |
| Model 6 | 0.05 | -309.4923 | -283.2060 | 111 172 |

**Table S4.** Structural and energetic parameters of the six preselected models after 100-ns MD simulations, including RMSD of disulfide bonds, binding free energies (MM/GBSA and MM/PBSA), and total atom count.

| Model | RMSD disulfide bonds | Binding Free Energy of the Complex | | Total atom count |
| --- | --- | --- | --- | --- |
|  |  | GB | PB |  |
| Model 3 | 0.04 | -297.05 | -304.72 | 91 106 |
| Model 5 | 0.04 | -222.51 | -223.56 | 146 876 |
| Model 6 | 0.05 | -448.75 | -312.62 | 111 172 |

**Table S5.** Structural and energetic parameters of the six preselected models after 1 µs MD simulations, including RMSD of disulfide bonds, binding free energies (MM/GBSA and MM/PBSA), and total atom count.

|  | **RMSF (Å)** | | |
| --- | --- | --- | --- |
| **Residue** | **Model 3** | **Model 5** | **Model 6** |
| **56** | 0.9713 | 0.8523 | 1.7434 |
| **129** | 1.2386 | 0.8321 | 2.0521 |
| **130** | 1.1950 | 0.8265 | 1.9463 |
| **945** | 1.0646 | 0.7123 | 1.9099 |

**Table S6.** RMSF of residues in the binding site.

| **Weighting Scheme** | **Model 3** | **Model 5** | **Model 6** |
| --- | --- | --- | --- |
| Energy – 0.20, RMSD – 0.15, RMSF – 0.15, Hydrogen bonds in A and C chains – 0.20, Hydrogen bonds in B and D chains – 0.20, Distance for chain 1 – 0.05, Distance for chain 2 – 0.05 | 0.86 | 0.34 | 0.43 |
| Energy – 0.25, RMSD – 0.25, RMSF – 0.10, Hydrogen bonds in A and C chains – 0.15, Hydrogen bonds in B and D chains – 0.15, Distance for chain 1 – 0.05, Distance for chain 2 – 0.05 | 0.90 | 0.31 | 0.45 |
| Energy – 0.30, RMSD – 0.20, RMSF – 0.10, Hydrogen bonds in A and C chains – 0.10, Hydrogen bonds in B and D chains – 0.10, Distance for chain 1 – 0.10, Distance for chain 2 – 0.10 | 0.89 | 0.26 | 0.55 |
| Energy – 0.10, RMSD – 0.20, RMSF – 0.20, Hydrogen bonds in A and C chains – 0.15, Hydrogen bonds in B and D chains – 0.15, Distance for chain 1 – 0.10, Distance for chain 2 – 0.10 | 0.83 | 0.38 | 0.38 |
| Energy – 0.20, RMSD – 0.20, RMSF – 0.10, Hydrogen bonds in A and C chains – 0.15, Hydrogen bonds in B and D chains – 0.15, Distance for chain 1 – 0.10, Distance for chain 2 – 0.10 | 0.90 | 0.28 | 0.48 |
| Energy – 0.10, RMSD – 0.25, RMSF – 0.15, Hydrogen bonds in A and C chains – 0.20, Hydrogen bonds in B and D chains – 0.20, Distance for chain 1 – 0.05, Distance for chain 2 – 0.05 | 0.87 | 0.39 | 0.33 |
| Energy – 0.15, RMSD – 0.30, RMSF – 0.15, Hydrogen bonds in A and C chains – 0.15, Hydrogen bonds in B and D chains – 0.15, Distance for chain 1 – 0.05, Distance for chain 2 – 0.05 | 0.87 | 0.38 | 0.35 |

**Table S7.** Composite scores of Models 3, 5, and 6 under different weighting schemes.
